# Multidimensionality of hallucination-like experiences: A factor structure refinement of the Launay-Slade Hallucination Scale

**DOI:** 10.1016/j.scog.2025.100368

**Published:** 2025-05-24

**Authors:** H. Honcamp, L.K. Goller, M. Amorim, S.X. Duggirala, J.F. Johnson, M. Schwartze, A.P. Pinheiro, S.A. Kotz

**Affiliations:** a Department of Neuropsychology and Psychopharmacology; Faculty of Psychology and Neuroscience, Maastricht University, the Netherlands; b Centro de Investigação em Ciência Psicológica, Faculdade de Psicologia, Universidade de Lisboa, Lisboa, Portugal; c Université Libre de Bruxelles, Belgium

**Keywords:** Launay-Slade Hallucination Scale, Exploratory factor analysis, Hallucination proneness, Psychosis continuum, Externalization bias, Risk assessment

## Abstract

**Background:**

Previous research on the multidimensionality of hallucination-like experiences (HLEs) across the psychosis continuum highlights methodological disparities, emphasizing the need for a cautious interpretation of findings and transparent reporting of parameters used in the analysis.

**Methods:**

This study aimed to refine the factorial structure of the 16-item Launay-Slade Hallucination Scale (LSHS), enhance methodological clarity, and improve the robustness of LSHS factor solutions. To this end, an Exploratory Factor Analysis (EFA) was performed on a heterogeneous sample (*N* = 278) with specified parameters (e.g., estimation procedure) that remain true to data characteristics and assumptions underlying EFA.

**Results:**

The results revealed a four-factor structure including “Multisensory HLEs”, “Auditory daydreaming”, “Vivid thoughts and inner speech”, and “Personified HLEs”. Our investigation introduces a new factor specific to the perceived presence of another person or another voice. This aligns with theories on self-monitoring difficulties associated with an external attribution bias as hallucination proneness (HP) increases across the continuum.

**Conclusion:**

The current results provide an opportunity for investigating neurophysiological and neurobehavioral correlates of HP considering highly differentiated individual profiles of HLEs. Future studies should focus on validating the robustness of the four-factor structure derived from this research across diverse samples of the general population (e.g., different age groups and cultural backgrounds). Specified composite scores underlying HLEs could be of additive value when assessing emerging clinical risk on the psychosis continuum.

## Highlights

• Methodological inconsistencies in acquiring LSHS factor structures are outlined.
• A transparent “true-to-data” EFA of LSHS data was performed on a non-clinical sample.
• The multidimensionality of HP is highlighted.
• The new “Personified HLEs” factor implies the perceived presence of another person.
• Refined factors might be more representative of individual differences in HLEs.

## Graphical abstract

**Figure:**
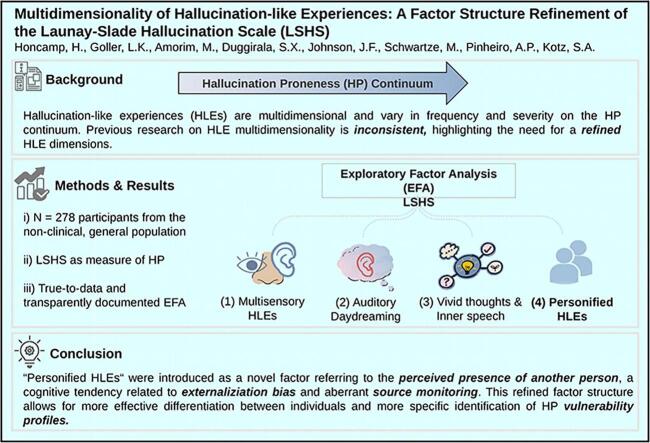

## 1 Introduction

Hallucination-like experiences (HLEs) are typically defined as untriggered and transitory sensory perceptions (Baumeister et al., 2017; Van Os, 2003). In the general population, HLEs vary in frequency and severity, and their persistence is associated with clinical risk, which is captured by the hallucination proneness (HP) continuum (Beavan et al., 2011; Linszen et al., 2022; Johns, 2005; Johns and Van Os, 2001). Moreover, there are considerable qualitative inter-individual differences in the subjective experience of HLEs and symptom affinities across sensory modalities. While some individuals tend to predominantly experience intrusive or vivid daydreams, others may report internal dialogues and voice-hearing (Linszen et al., 2022; Preti et al., 2014). This suggests that HP is not a single dimension but a multifaceted construct with distinct symptom profiles.

The Launay-Slade Hallucination Scale (LSHS; (Launay and Slade, 1981)) is a widely used self-report questionnaire to assess HP across clinical and non-clinical populations (see supplementary material A). Moreover, the LSHS has proven to be a useful tool in assessing the multidimensionality of HP by means of Exploratory Factor Analysis (EFA) or Principal Component Analysis (PCA; (Vellante et al., 2012; Serper et al., 2005; Fonseca-Pedrero et al., 2010)). The resulting factors or principal components are thought to reflect different HP dimensions and provide a means to further differentiate inter-individual variability in HLEs. However, substantial differences in participant characteristics, item inclusion and modification, response format, and analytic decisions reduce comparability between proposed factor structures, and render the interpretation of findings across studies more difficult (Smailes et al., 2022). In particular, the use of divergent analysis parameters, such as varying factor extraction methods, rotation techniques, and inconsistent reporting of statistical assumptions (e.g., multivariate normality, sampling adequacy), complicate the interpretation of findings across studies. Additionally, some studies employed PCA while referring to it as EFA, despite the conceptual and statistical distinctions between these approaches (Suhr, 2005; Watkins, 2018) (Table 1). These inconsistencies undermine the reliability and generalizability of previously reported factor solutions.

**Table 1:** Overview of study differences in LSHS implementation and derived factor structures.

Study	N	Sample characteristics^a^	LSHS response format	LSHS items and modifications	Analysis	Derived factor structure
Launay and Slade (1981)	296	*Normal control group* (*n* = 54, 24 male, 30 female, mean age = 33.67, SD = 7.78) *Patient group* (*n* = 42, 23 male, 19 female, mean age = 40.12, SD = 13.24) *Prisoner group* (*n* = 200, 100 male, 100 female, mean age = 23.51, SD = 7.99)	Binary (True-false)	30 in total, including three miscellaneous items (e.g. “I seldom think about the future at all”)	Endorsement frequencies per item were contrasted between normal control and patient group by using chi-square tests Patients scored higher on 16 items. These were further analyzed based on the entire sample (*N* = 296) with an unrotated PCA (SPSS package PA2), resulting in a 12 item solution	2-factor structure: “tendency to hallucinatory experiences” and “negative response set”
Bentall and Slade (1985)	150 (136 completed LSHS-A and 118 of them completed LSHS-B)	Male undergraduate students (mean age = 19.8, SD = 1.2)	5-point Likert scale *(“certainly applies”…”certainly does not apply”*)	LSHS-A: original version derived from Launay & Slade (1981) with two negative response items (9 and 11) changed into positive ones LSHS-B: similar scale, slightly reworded (but not validated) and in a different order	NA	NA
Levitan et al. (1996)	169	Psychiatric patients^b^ with a documented history of auditory hallucinations	5-point Likert scale *(“certainly applies”…”almost always”*)	Modified 12-item version of LSHS as used in Bentall & Slade (1985)	PCA with Varimax rotation (orthogonal)	4-factor structure: “vivid daydreams”, “clinical auditory hallucinations”, “intrusive/vivid thoughts”, “sub-clinical auditory hallucinations” (perceptual aberrations)
Morrison et al. (2000)	105	Normal participants: Undergraduate students or health professionals (21 male, 84 female, mean age = 30.4, age range = 20–57, SD = 9.3)	4-point Likert scale *(“never”…”certainly does not apply”, no neutral response option*)	16-item version of LSHS (12-items from original version (Launay & Slade, 1981) plus three additional items on visual hallucinations, remaining item is not further specified)	PCA with Oblimin (oblique) rotation (based on 15 items, as one items was never endorsed by any of the participants)	2-factor structure: “predisposition towards visual hallucinations/disturbances” and “tendency to experience auditory or verbal hallucinations/daydreaming”
Aleman et al. (2001)	243	Undergraduate students (58 male, 185 female, mean age = 22.6, SD = 5.6)	5-point Likert scale *(“certainly applies”…”certainly does not apply”*)	Dutch translation of 12-item LSHS version by Bentall & Slade (1985)	PCA with oblique rotation (Oblimin with Kaiser normalization)	3-factor structure: “tendency towards hallucinatory experiences”, “subjective externality of thought”, and “vivid daydreams”
Morrison et al. (2002)	132	Health service employees, volunteers (39 male, 93 female, mean age = 35.19, SD = 10.71, range = 19–59)	4-point Likert scale (“never”…”almost always”)	Revised hallucination scale (RHS), 24-item revised version of LSHS version by Bentall & Slade (1985) Modifications: Additional items on visual hallucinations, predisposition to auditory hallucinations, vividness of imagery, and daydreaming	PCA with oblique rotation (oblimin)	3-factor structure: “vividness of imagination and daydreaming”, “tendency to experience visual disturbances and hallucinations”, and “tendency towards experiencing auditory hallucinations”
Waters et al. (2003)	562	Undergraduate students (176 males, 386 females, mean age = 18.79, SD = 4.03)	5-point Likert scale *(“certainly applies”…”certainly does not apply”*)	“LSHS-R" (12-item LSHS version) as revised in Bentall & Slade (1985)	PCA with oblique rotation (Oblimin with Kaiser normalization)	3-factor structure: “vivid mental events”, “hallucinations with a religious theme", and “auditory and visual hallucinatory experiences”
Larøi et al., 2004a, Larøi et al., 2004b	265	Normal population (72 male, 193 female, mean age = 23.7, SD = 8.6)	5-point Likert scale *(“certainly applies”…”certainly does not apply”*)	French translation of a modified, 17-item version of the LSHS Four modifications within this study: 1. including visual items by Morrison et al. (2000) 2. including items from tactile and olfactory modality 3. including an item referring to the experience of the presence of someone close who has passed away, excluding items (e.g. “I have heard the voice of the devil”) 4. including two more items referring to hypnagogic and hypnopompic hallucinations	PCA with Varimax rotation with normalization (orthogonal)	4-factor structure: “sleep-related hallucinatory experiences”, “vivid daydreams”, “intrusive thoughts or realness of thought”, and “auditory hallucinations”
Larøi and Van Der Linden (2005)	236	Non-clinical subjects: University students (59 % male, 41 % female, mean age = 22.6, age range = 18–34, SD = 3.6)	5-point Likert scale *(“certainly applies”…”certainly does not apply”*)	Modified 16-item LSHS version as used by Larøi et al. (2004) Two modifications within this study: 1. Due to low response rate, the item “In the past, I have heard the voice of God or one of his messengers speaking to me" was removed. 2. A visual hallucination items was replaced by another	PCA with Varimax rotation (orthogonal)	5-factor structure: “sleep-related hallucinatory items”, “vivid daydreams”, “intrusive or vivid thoughts”, “auditory hallucinations”, and “visual hallucinations”
Serper et al. (2005)		*Schizophrenic patients with evidence of auditory hallucinations* (*n* = 39, 88 % male, mean age = 34.8, SD = 12.2) *Schizophrenic patients without active hallucinations* (*n* = 49, 91 % male, mean age = 33.4, SD = 11.9) *Sample of additional undergraduate students* (*n* = 363, 48 % male, mean age = 19.5, SD = 1.5)	5-point Likert scale *(“certainly applies”…”certainly does not apply”*)	LSHS-A (Bentall & Slade, 1985)	Principal axis factor analysis performed separately for each of the three groups with Promax rotation (oblique)	2-factor structure for all conditions: “subclinical” and “clinical”
Fonseca-Pedrero et al. (2010)	807	University students (245 males, 562 females, mean age = 20.19, range = 17–32, SD = 2.98)	5-point Likert scale *(“certainly applies”…”certainly does not apply”*)	“LSHS-R" (Bentall & Slade, 1985)	PCA with direct oblimin rotation (oblique)	2-factor structure: “hallucinatory experiences” and “vivid mental events”
Vellante et al. (2012)	437	Young adults (178 male, mean age = 24.7, range = 18–34, SD = 3.4; 259 female, mean age = 24.7, range = 18–45, SD = 3.5)	5-point Likert scale *(“certainly applies”…”certainly does not apply”*)	16-item version of LSHS as used in Larøi & Van der Linden (2005)	EFA was carried out using FACTOR (Lorenzo-Seva and Ferrando, 2006) with Promax rotation (oblique)	4-factor structure: “auditory and visual hallucinatory experiences”, “multisensory hallucinatory experiences”, “intrusive thoughts”, and “vivid daydreams”
Preti et al. (2014)	649	Non-clinical population (305 males, 344 females, mean age = 24, SD = 3.4)	5-point Likert scale *(“certainly applies”…”certainly does not apply”*)	16-item version of LSHS as used in Larøi & Van der Linden (2005) Here: also termed the “LSHS-E" (extended)	CFA with previous factor structures	4-factor structure: “intrusive thoughts”, “vivid daydreams”, “multisensory hallucination like experiences”, and “auditory and visual hallucination like experiences”
Castiajo and Pinheiro (2017)	354	College students (88 male, 266 female, mean age = 24.13, range = 18–64, SD = 6.61)	5-point Likert scale *(“certainly applies”…”certainly does not apply”*)	16-item version of LSHS as used in Larøi & Van der Linden (2005)	PCA with Varimax rotation (orthogonal)	3-factor structure: “auditory, visual, olfactory, and tactile hallucinations”, “vividness of daydreams”, and “intrusive or vivid thoughts and sleep-related hallucinations”
Siddi et al. (2018)	422	*Individuals with verified psychotic disorders* (*n* = 111, 38 males, 73 females, mean age = 40.91, SD = 11.68) *Healthy controls* (*n* = 311, 85 male, 226 female, mean age = 37.49, SD = 10.89)	5-point Likert scale *(“certainly applies”…”certainly does not apply”*)	16-item version of LSHS as used in Larøi & Van der Linden (2005) Here: also termed the “LSHS-E" (extended)	CFA with previously established factor structures (unidimensional model, the 2-factor structure by Serper et al. (2005), and the 4-factor structure by Vellante et al. (2012)) According to authors, 3-factor structures were not tested, as those were derived from studies implementing the 12-item version of the LSHS	4-factor structure: “intrusive thoughts”, “vivid daydreams”, “multisensory hallucination like experiences”, and “auditory visual hallucination like experiences”
Sahu et al. (2020)	164	General population: (range = 18–65)	5-point Likert scale *(“certainly applies”…”certainly does not apply”*)	Hindi translation of the 16-item version of LSHS as used in Larøi & Van der Linden (2005)	CFA with previous factor structures (unidimensional model, the 2-factor structure by Serper et al. (2005), and the 4-factor structure by Vellante et al. (2012) and Siddi et al. (2018)	4-factor structure: “intrusive thoughts”, “vivid daydreams”, “multisensory hallucination like experiences”, and “auditory visual hallucination like experiences”

a Condition labels and all sample characteristics are reported as in the corresponding publication.

b No further description of sample characteristics; Not applicable (NA), Launay-Slade Hallucination Scale (LSHS), Principal component analysis (PCA), Exploratory factor analysis (EFA), Confirmatory factor analysis (CFA).

Moreover, HLEs across non-clinical and clinical groups are most often documented in the auditory domain (García-Montes et al., 2006; Larøi et al., 2012; Slotema et al., 2012; Waters and Fernyhough, 2017). This justifies addressing the neurophysiological underpinnings of the proneness to auditory HLEs using a composite score derived from “auditory” LSHS items (Pinheiro et al., 2020; Honcamp et al., 2024). However, the affinity towards HLEs can be multisensory. For example, the factor structure proposed by Castiajo and Pinheiro (Castiajo and Pinheiro, 2017) revealed three factors, with one of them referring to auditory, visual, olfactory, and tactile hallucinations. Consequently, modality-specific composite scores may not adequately describe the multidimensionality and heterogeneity of HLEs across the HP continuum.

Given the methodological heterogeneity in previously proposed factor solutions, the current two-center study aimed to provide a more representative and robust factor structure, based on transparent methodological choices that remain true to data characteristics and assumptions underlying EFA (Watkins, 2018; Goretzko et al., 2021). Considering that HP constitutes an important dependent variable in research on the cognitive and neurophysiological mechanisms of HLEs, a more methodologically justified factor solution should yield more robust composite scores that allow for a refined assessment of inter-individual differences and vulnerability profiles.

## 2 Material and methods

### 2.1 Participants and procedure

A group of 278 participants (206 females, 71 males, 1 other; mean age = 22.47, age SD = 4.48, age range: 18–48) participated in this study. Participants were recruited at Maastricht University, the Netherlands, and the University of Lisbon, Portugal, through social media advertisements, via e-mail, and university-specific research participation systems. The LSHS was administered as an online questionnaire. The English and Portuguese adaptations of the LSHS, administered as an online questionnaire, were used to assess HP (Castiajo and Pinheiro, 2017; Larøi and Van Der Linden, 2005). The current version of the LSHS consists of 16 items that are answered on a 5-point Likert scale ranging from 0 to 4 (0 = “*definitely does not apply to me*,” 1 = “*possibly does not apply to me*,” 2 = “*unsure*,” 3 = “*possibly applies to me*,” and 4 = “*definitely applies to me*"). Higher scores indicate higher HP. The distribution of scores across all response categories and all items can be found in Supplementary material B1, Supplementary Table 1. All participants provided written informed consent before participation. Ethical approval for the study was granted by the Ethics Review Committee Psychology and Neuroscience (ERCPN) at Maastricht University (ERCPN-176_08_02_2017), the Dutch Medisch-ethische toestingscommissie (METC 20-035), and the Deontological Committee of Faculty of Psychology at the University of Lisbon.

### 2.2 Exploratory factor analysis

To ensure transparency and clarity in methodological choices, the following sections outline the specific procedures used in this study. Any deviations from standard practices, along with justifications for these modifications can be found in Supplementary Material B2.

#### 2.2.1 Data suitability

Multivariate normality of the data was tested using the Mardia Test and the Henze-Zirkler Test (Henze and Zirkler, 1990; Mardia, 1970), implemented in the R *mvnormalTest* package. Both tests revealed a violation of multivariate normality, which was confirmed by inspection of individual histograms for each item. We therefore opted for polychoric correlations in evaluating data suitability for EFA and factor estimation. Inspection of the polychoric correlation matrix revealed that inter-item correlation coefficients were reasonably high (Supplementary Fig. 1). No item was removed, thus the EFA was performed on all 16 items (Supplementary Material B2.1). Data suitability for EFA was further assessed using the Kaiser-Meyer Olkin (KMO) criterion (overall KMO = 0.824) and Barlett's test of sphericity. The KMO assesses sampling adequacy based on the common variance of all items and returned an overall value of 0.824 (Supplementary Table 2). Values above 0.8 are considered meritorious/good (Howard, 2016). Barlett's test was significant (Chi-square = 1956.7, *p* < 0.001), indicating that all correlation coefficients are significantly different from zero. Lastly, although there is no strict sample size requirement for EFA, the current sample size (*N* = 278) and participant-to-variable ratio (17.4:1) are in line with common recommendations, i.e., minimum sample size of 200 and a minimum of five participants for each included variable (Howard, 2016).

#### 2.2.2 Factor extraction

The EFA (common factor analysis) was performed in R version 4.3.2 using the *lavaan* package version 0.6–16 (Rosseel, 2012). We used the robust mean and variance-adjusted weighted least squares (WLSMV) method for factor estimation (Supplementary Material B2.2). The R *lavaan* package allows explicit specification of the data format as ordinal and accordingly bases the factor estimation on the polychoric correlations matrix. We further opted for promax oblique rotation as it better reflects simple structure and accounts for the assumption of correlated latent psychological constructs (Goretzko et al., 2021; Fabrigar et al., 1999; Hendrickson and White, 1964; Finch, 2006; McLeod et al., 2001).

#### 2.2.3 Factor retention criteria

The most accurate factor solution was derived by a combination of commonly used factor retention criteria, including the Kaiser-Guttman Criterion (KGC, eigenvalues ≥1), the Scree test, the lower bound of the RMSEA 90 % confidence interval, and the interpretability of factors. The KGC and the Scree plot seemed to point towards retaining five factors; however, the fifth eigenvalue barely exceeded 1 (Supplementary Fig. 2; Supplementary Table 3). This indicates that this criterion may not be strong enough to justify the retention of all five factors (Supplementary Material B 2.3). To further inform this decision, a parallel analysis was conducted using both PCA- and EFA-based eigenvalues. Interestingly, the PCA-based parallel analysis suggested a three-factor solution, while the EFA-based version supported retaining five factors. These divergent results illustrate the sensitivity of factor retention decisions to the underlying assumptions of the method used. In this context, the four-factor solution may be viewed as a theoretically and empirically grounded compromise. This aligns with the lower bound of the RMSEA 90 % confidence interval, which is recommended as the criterion of choice if the overall goal is to seek a model that best approximates the data, i.e., maximizing accuracy (Preacher et al., 2013). According to this criterion, a four-factor solution was preferred for the current data set. Finally, inspection of the rotated four- and five-factor pattern coefficients revealed that the five-factor solution was less interpretable due to an increased number of items with cross-factor loadings (i.e., loadings ≥ | 0.3 | on more than one factor; Supplementary Table 5), indicating that the presence of the complex item in the four-factor solution is likely not due to under-factoring (Watkins, 2018). We therefore decided to retain four factors.

## 3 Results

### 3.1 Exploratory factor analysis - factor solution

The rotated factor loadings (i.e., pattern coefficients) and communalities of all items can be found in Table 2. The first factor was labeled “Multisensory HLEs” and consisted of five items referring to hypnagogic, hypnopompic, olfactory, visual, and tactile hallucinatory experiences. The second factor, labeled “Auditory daydreaming” included two items that assess the vividness of sounds while daydreaming. Factor three comprised six items related to the heightened vividness of thought (e.g., “Sometimes my thoughts seem so real as actual events in my life”) and inner speech (“I often hear a voice speaking my thoughts aloud”) and was therefore labeled “Vivid thoughts and inner speech”. Finally, factor four consisted of items that explicitly refer to another person (e.g., “On certain occasions, I have had the feeling of the presence of someone close who has deceased”, and “In the past, I have had the experience of hearing a person's voice and then found that no one was there") and was labeled “Personified HLEs”. The inter-factor correlations, internal consistency estimates (Cronbach's Alpha), and the cumulative explained variance are summarized in Table 2. The four extracted factors together explained 52.8 % of the total variance.

**Table 2:** Rotated factor loadings (pattern coefficients) and item communalities.

LSHS items	F1	F2	F3	F4	Comm.
1. I have had the feeling of touching something or being touched and then found that nothing or no one was there.	**0.78**	0.10	−0.03	−0.09	0.56
2. Sometimes, immediately prior to falling asleep or upon awakening, I have had a sensation of floating or falling or that I left my body temporarily.	**0.47**	−0.02	0.29	−0.26	0.34
3. Sometimes, immediately prior to falling asleep or upon awakening, I have had the experience of having seen or felt or heard something or someone that wasn't there or the feeling of being touched even though no one was there.	**0.82**	−0.03	−0.22	0.10	0.53
4. On certain occasions, I have had the feeling of the presence of someone close who has deceased.	0.11	0.11	−0.11	**0.41**	0.22
5. In my daydreams, I can hear the sound of a tune almost as clearly as if I were actually listening to it.	0.20	**0.85**	−0.02	−0.21	0.77
6. The sounds that I hear in my daydreams are usually clear and distinct.	−0.09	**0.80**	0.10	0.03	0.67
7. The people in my daydreams seem so true to life that sometimes I think that they are.	−0.24	0.21	**0.66**	0.18	0.52
8. Sometimes my thoughts seem as real as actual events in my life.	−0.22	0.09	**0.97**	0.04	0.77
9. No matter how hard I try to concentrate, unrelated thoughts always creep into my mind.	0.28	−0.10	**0.60**	−0.23	0.46
10. Sometimes a passing thought will seem so real that it frightens me.	0.09	−0.13	**0.72**	−0.06	0.52
11. I have been troubled by hearing voices in my head.	0.09	−0.09	**0.43**	**0.33**	0.51
12. In the past, I have had the experience of hearing a person's voice and then found that no one was there.	0.23	−0.11	0.12	**0.47**	0.47
13. I often hear a voice speaking my thoughts aloud.	0.14	0.01	**0.41**	0.06	0.32
14. On certain occasions, I have seen the face of a person in front of me, but then there was no one.	−0.06	−0.11	0.00	**1.06**	0.99
15. Sometimes I have seen things or animals when nothing was in fact there.	**0.51**	0.16	−0.03	0.20	0.48
16. In the past, I have smelled a particular odor when there was nothing there.	**0.31**	0.11	0.03	0.22	0.30
Cumulative expl. variance (%)	17.3	30.9	43.0	52.8	
Cronbach's Alpha	0.68	0.77	0.76	0.60	
Inter-factor correlations					
F1	1.00				
F2	0.37⁎⁎	1.00			
F3	0.74⁎⁎	0.39⁎⁎	1.00		
F4	0.61⁎⁎	0.29⁎⁎	0.54⁎⁎	1.00	

*Note.* LSHS item order was adapted from Laroi and van der Linden (2005). Threshold for salient items in bold = |0.30|. F1 = “Multisensory HLEs”, F2 = “Auditory Daydreaming”, F3 = “Vivid thoughts and inner speech”, F4 = “Personified HLEs”, Comm. = Communalities.

⁎⁎ *p* < 0.01.

### 3.2 Goodness-of-fit

The exact Chi-square model fit test (null hypothesis of no difference between the model-implied covariance matrix and the actual observed covariance matrix) supported accepting the model as “fitting” (i.e., X2 = 62.365, df = 62.0, *p* = 0.535; (Barrett, 2007)). Since the Chi-square statistic is sensitive to sample size (Brown, 2015), the following additional (scaled) goodness-of-fit statistics were used to evaluate the model fit: the scaled comparative fit index (CFI), scaled RMSEA and corresponding 90 % confidence interval (CI), and the standardized root mean square residual (SRMR). The CFI builds upon a comparison with a baseline model (i.e., an independence model, in which all error variances are fixed to zero and all factor loadings are fixed to 1). The scaled CFI was equal to 0.973, surpassing the 0.97 threshold indicating a good fit to the model (Schermelleh-Engel et al., 2003). RMSEA is a measure of model fit by approximation. Values lower than or equal to 0.05 reflect a “close fit” and values between 0.05 and 0.08 are considered “adequate” (Schermelleh-Engel et al., 2003). Additionally, the RMSEA CI provides an assessment of the precision of the point estimate. The scaled RMSEA for the four-factor solution was 0.057 [CI 0.042 0.073]. The SRMR based on fitted residuals of the current model was 0.045, indicating an acceptable value below the 0.50 fitness threshold (Brown, 2015). In summary, all fit measures indicated an adequate to close model fit. Note, that the conventional cut-off values for the above goodness-of-fit statistics are based on normally distributed, continuous data with ML estimation, and may therefore differ from the true model fit resulting from WLSMV estimation with polychoric correlations (Xia and Yang, 2019). However, the SRMR point estimate yields acceptable type 1 error rates, thus it may be more powerful for ordinal data than the RSMEA (Shi et al., 2020).

## 4 Discussion

The current study investigated the factorial structure of the 16-item LSHS (Larøi and Van Der Linden, 2005; Larøi et al., 2004a) to establish a more robust, differentiated, and reliable factor solution in a heterogeneous and international sample. Previous research on LSHS-based EFA and PCA was reviewed and methodological differences between studies were identified in relation to sample characteristics, LSHS response format, item inclusion and modifications, type of analyses, and derived factor structures. An EFA was performed with carefully chosen parameters, considering data characteristics and assumptions underlying EFA estimation procedures (i.e., ordinal items, non-normality). The analysis revealed a four-factor solution including “Multisensory HLEs”, “Auditory daydreaming”, “Vivid thoughts and inner speech”, and “Personified HLEs”.

### 4.1 Comparison of current EFA-derived factor structure with previous studies

Two aspects deserve special attention when discussing the results. First, participant groups in the referred literature not only differ in HP (ranging from clinical to non-clinical subsamples) but also in their demographics (e.g., age and education). Second, some previously reported dimension reduction solutions were based on the 12-item version of the LSHS (Serper et al., 2005; Fonseca-Pedrero et al., 2010; Aleman et al., 2001; Waters et al., 2003), used a different response format (i.e., 4, instead of 5-point Likert scale, Morrison et al., 2000), or different item combinations and modifications (Larøi and Van Der Linden, 2005; Larøi et al., 2004a; Morrison et al., 2000). This means that direct comparisons between previous and current results might not be feasible (Table 1). The following discussion therefore centers on factor solutions derived from the extended 16-item, 5-point Likert scale LSHS, including the originally proposed five-factor model (Larøi and Van Der Linden, 2005) and alternatively suggested three- (Castiajo and Pinheiro, 2017) and four-factor structures (Vellante et al., 2012).

#### 4.1.1 Multisensory HLEs

The first “Multisensory HLEs” factor consisted of five items that refer to hypnagogic, hypnopompic, olfactory, visual, and tactile hallucinatory experiences, which is partly consistent with previous results. Hypnagogic and hypnopompic hallucinations were grouped together with tactile hallucinations in a five-factor structure (Larøi and Van Der Linden, 2005) and with both tactile and olfactory hallucinations in a four-factor structure (Vellante et al., 2012). In contrast, the three-factor structure proposed by Castiajo and Pinheiro (Castiajo and Pinheiro, 2017) revealed a factor combining sleep-related, daydreaming, and vivid thought items. While some evidence suggests a relationship between hypnagogic, hypnopompic hallucinations, and intrusive thoughts (McCarthy-Jones et al., 2011), the formulation of items 2 and 3 (“feeling of being touched”, “sensation of floating or falling”) seems to support the replicated association with tactile HLEs. Likewise, the item formulations within the “Multisensory HLEs” differs from all other items in that they refer to a collection of hallucinatory experiences connected by a non-mutually exclusive “or” (e.g., touching someone *or* being touched; seen objects *or* animals; seen, felt, *or* heard something *or* someone). Thus, compared to items with a simpler and more distinguished phrasing, these items cover a broader range of HLEs and warrant ambiguous interpretations.

#### 4.1.2 Auditory daydreaming

LSHS items 5 (“In my daydreams, I can hear the sound of a tune almost as clearly as if I were actually listening to it”) and 6 (“The sounds that I hear in my daydreams are usually clear and distinct”), formed factor 2 “Auditory daydreaming”. The co-occurrence of items 5 and 6 within the same factor is consistent with previous literature. However, these items were usually grouped together with additional items (e.g., those referring to vivid thoughts and auditory HLEs). Larøi and Van Der Linden (Larøi and Van Der Linden, 2005) discovered a “Vivid daydreams” factor that included items 5, 6, 7, and 8, thus representing a more generalized vividness of thoughts. Similarly, the “Vivid daydreams” factor identified by (Vellante et al., 2012) consisted of items 5, 6, 7, and 12. Whereas items 5, 6, and 7 explicitly refer to the experience of daydreaming, item 12 (“In the past, I have had the experience of hearing a person's voice and then found that no one was there") has little semantic overlap with the other items. The “Auditory daydreaming” factor in the current factor solution slightly differs from the above in that it consists of only two items that explicitly refer to the vividness of sounds. Although it was suggested that a factor containing less than three salient items may be an indication of an over-factored EFA solution (Watkins, 2018), this factor remained consistent across alternatively considered models with three and five factors (see Supplementary material C). The formation of the “Auditory daydreaming” factor might thus be a consequence of diligently chosen model estimation parameters that are in line with underlying assumptions and data characteristics and not a result of model misspecification. The current results further suggest that HLEs within the auditory modality may not necessarily cluster together, i.e., the false perception of sounds (items 5 and 6) may be qualitatively different from voice and inner speech-related hallucinatory percepts (items 11, 12, and 13).

#### 4.1.3 Vivid thoughts and inner speech

Items combined in the “Vivid thoughts and inner speech” factor referred to vivid daydreaming (items 7 and 8), intrusive thoughts (item 10), and vivid inner speech (items 9 and 11). Most of these items explicitly broached the issue of differentiating imagination from reality, irrespective of the sensory domain. This is in line with theories on self- and reality-monitoring difficulties along the HP continuum (Brookwell et al., 2013; Damiani et al., 2022; Woodward et al., 2007) where HLEs can be linked to externalization bias (Brookwell et al., 2013; Frith, 1987). In contrast, previously proposed factor structures commonly clustered auditory and visual HLEs in the same factor, supporting the multidimensionality of HP across sensory domains. Vellante, Larøi (Vellante et al., 2012) found an “Auditory and visual HLEs” factor comprising items 11, 13, and 14. In the current EFA solution, items 11 and 14 are also loaded on the same factor (factor 4), together with items 4 and 12, referring to the sensed presence of a deceased person and the perception of another person's face. The original five-factor factor solution distinguished between an “Auditory hallucinations” and a “Visual hallucinations” factor (Larøi and Van Der Linden, 2005), whereas Castiajo and Pinheiro (Castiajo and Pinheiro, 2017) found a more extended factor that combines auditory, visual, olfactory, and tactile HLEs. This inconsistency could reflect that factor solutions with a higher or lower number of factors are more “fine-grained” or “coarse” by default (Watkins, 2018).

#### 4.1.4 Personified HLEs

In contrast to earlier factor structures, the current EFA model revealed a factor, “Personified HLEs”, which includes items implicating the presence or appearance of another person, e.g., the false perception of the presence of another person (item 4), another person's voice (item 12), and another person's face (item 14). The overarching theme of “Personified HLEs” is emphasized even more upon comparing this factor with factor 1 (“Multisensory HLEs”): whilst both factors comprise items with a similar sentence structure that refer to a sensory experience (touch, vision, audition, and smell) although, in fact, *nothing was there,* items from the “Personified HLEs” factor explicitly refer to another person's presence. This is not the case for the “Multisensory HLEs” factor, which also includes experiences related to e.g., animals (item 15). Furthermore, items 1 and 3 indeed refer to sensory experiences relating to “someone” or “something” being there, although “no one" or “nothing” was there. However, these items could be interpreted ambiguously. Therefore, a theme that is purely referring to the person component within HLEs seems to be a unique characteristic of factor 4.

Item 11 (“I have been troubled by hearing voices in my head”), which, in turn, also loaded on the “Vivid thoughts and inner speech” factor was also included in factor 4. Looking closely at the formulation of item 11 in relation to all other items of factor 3 and 4, we suggest that item 11 could be interpreted differently depending on the individual's overall hallucinatory vulnerability and (clinical) risk. In other words, low-risk individuals may experience vivid inner speech and/or intrusive thoughts as ambiguous (i.e., imagination versus reality) but successfully attribute them to an internal source, whereas high-risk individuals may tend to attribute HLEs to an external source, like another person (Allen et al., 2006; Larøi et al., 2004b). This idea is in line with the external attribution bias observed in hallucinating and hallucination-prone individuals (Brookwell et al., 2013; Levine et al., 2004). Consequently, scores on factors 3 and 4 may help differentiating between individuals with more transient and benign HLEs and those with a heightened risk of developing clinically relevant hallucinations. This hypothesis should be systematically explored in future studies.

In summary, the current factorial structure enhances our conceptual understanding of HLEs across the HP continuum. While it partly overlaps with prior models (e.g., a similar Multisensory HLEs factor in Vellante, Larøi (Vellante et al., 2012)), it also reveals a novel Personified HLEs factor, capturing externalization bias, i.e., the tendency to attribute self-generated stimuli to external sources. While meta-analytic evidence supports deficits in source-monitoring and signal detection (Brookwell et al., 2013) other studies do not find supporting evidence for source monitoring difficulties in individuals with high HP (Garrison et al., 2017; McKague et al., 2012). The multidimensional framework of HLEs may clarify these inconsistencies by capturing inter-individual variability more directly. Importantly, the novel Personified HLEs factor may contribute to more robust and reliable composite scores reflecting individual symptom profiles in the general population.

### 4.2 Limitations and implications for future research

The four-factor structure proposed by Vellante, Larøi (Vellante et al., 2012) was frequently confirmed by means of Confirmatory Factor Analysis (CFA) when directly compared to alternative unidimensional, two-, three- or five- factor models (Table 1, (Preti et al., 2014; Siddi et al., 2018; Sahu et al., 2020)). Thus, it seems plausible that four factors adequately describe the HP multidimensionality, which is consistent with the current results. However, previously proposed EFA solutions have not been directly compared to alternative models with the same number of factors but distinct item combinations. Future studies should therefore investigate the goodness-of-fit of both the previously proposed and confirmed four-factor model (Vellante et al., 2012) and the four-factor model proposed in the current study. This is particularly important given the identification of a two-item factor (Auditory Daydreaming) in the current factor solution. Although acceptance of this factor is theoretically and empirically grounded, it is essential to assess whether it can be reliably identified in different independent samples. Verifying the current factor-structure's stability is a necessary next step and should ultimately support the generalizability of findings, given that stable structures render similar findings within comparable samples.

Moreover, future investigations should note that age may affect derived factor structures and could compromise the generalizability of results (Steenkamp et al., 2021; Yates et al., 2021). Furthermore, different modalities of HLEs seem to be predominant within different age groups (Larøi et al., 2019; Wehling et al., 2021). Therefore, a systematic exploration of hallucinatory vulnerability profiles and corresponding factor structures across different age groups seems necessary when investigating clinical risk in the general population.

Next to EFA, previously reported factor structures were also derived from PCA. While the purpose of EFA is to re-define measurement variables with a smaller number of latent constructs, the aim of PCA is data reduction by means of linear combinations of the original variables (Watkins, 2018; Gaskin and Happell, 2014). Hence, these approaches differ considerably in their underlying assumptions and derivation of factor and principal components scores (Suhr, 2005; Sürücü et al., 2022). Some previous studies also did not provide information about relevant data characteristics (e.g., normality; ordinal items), analyzed correlation matrix (e.g., Pearson's, Spearman's, polychoric), and EFA/PCA parameter choices including their respective assumptions (e.g., ML estimation assuming continuous multivariate-normally distributed data). Increased transparency and careful consideration of data characteristics in future EFA and CFA studies could enhance comparability and generalizability of factor structures and the suitability of resulting composite scores for research, as these are important when investigating individual differences. The present findings could provide a foundation for future research aimed at further establishing methodologically sound EFA standards, which render reliable factor structures. In context of HLE research, this could lead to a more refined understanding of qualitative characteristics of HLE across the general population.

Another critical point is that, despite its strong validation and widespread use, the LSHS may overemphasize cognitive and auditory experiences, underrepresenting visual, tactile, and especially olfactory and gustatory modalities. This could overlook important individual differences in HLEs across the HP continuum. The LSHS also focuses heavily on internal verbal experiences linked to higher HP, potentially underrepresenting the more multidimensional and subtle mixed-modality experiences common in non-clinical populations. Nevertheless, the LSHS version validated by Larøi and Van Der Linden (Larøi and Van Der Linden, 2005) was used to ensure methodological rigor and comparability, with the aim of refining its dimensional structure while preserving psychometric integrity.

Lastly, the current results offer an opportunity to further differentiate individual symptom and affinity profiles along the HP continuum. Many previous studies have addressed the neural underpinnings of modality-specific HP using composite scores derived from LSHS items relating to, e.g., auditory, auditory-verbal, or visual HLEs (Honcamp et al., 2024; Alderson-Day et al., 2022; El Haj et al., 2016; Morrison et al., 2000; Pinheiro et al., 2019). However, the current findings highlight that items subsumed under the auditory modality do not cluster together but are distributed across three of the four identified factors. This suggests that individual variability in HP may be better captured by composite scores derived from methodologically sound factor analysis, rather than sum-scores of individual items within one sensory modality. Future research should explore whether these more differentiated HP dimensions correspond to distinct neurophysiological underpinnings. For instance, the proneness to engage in auditory daydreaming might be linked to brain activity patterns similar to those seen during mind wandering, e.g., heightened activity in the default-mode network (Christoff, 2012), while personified HLEs could be associated with the neural mechanisms underlying error monitoring. This includes aberrant activity in the anterior cingulate and medial prefrontal cortices (Kowalski et al., 2021), altered oscillatory activity, and reduced suppression in response to self-generated stimuli (Pinheiro et al., 2018).

## 5 Conclusions

This study contributes to a better understanding of HP by scrutinizing the factor structure of the 16-item LSHS. The exploration of a diverse sample revealed a nuanced four-factor solution that comprised “Multisensory HLEs”, “Auditory daydreaming”, “Vivid thoughts and inner speech”, and “Personified HLEs”. The current results offer a distinct perspective that emphasizes the importance of a multisensory approach to HP and sheds light on the relationship between auditory daydreaming and vivid thoughts. Additionally, the identification of a factor specific to the perceived presence of another person introduces a novel perspective in understanding the multidimensionality of HLEs across the HP continuum. Moreover, this study highlights the necessity for transparency in EFA studies and alignment of analysis choices with underlying data characteristics to increase comparability and generalizability across studies.

## CRediT authorship contribution statement

**H. Honcamp:** Writing – review & editing, Writing – original draft, Visualization, Resources, Project administration, Methodology, Formal analysis, Data curation, Conceptualization. **L.K. Goller:** Writing – review & editing, Writing – original draft, Resources, Project administration, Methodology, Formal analysis, Data curation, Conceptualization. **M. Amorim:** Writing – review & editing, Resources. **S.X. Duggirala:** Writing – review & editing, Resources. **J.F. Johnson:** Writing – review & editing, Resources. **M. Schwartze:** Writing – review & editing, Supervision, Conceptualization. **A.P. Pinheiro:** Writing – review & editing, Supervision, Resources. **S.A. Kotz:** Writing – review & editing, Resources, Conceptualization.

## Ethics approval statement

Ethical approval was granted by Ethics Review Committee Psychology and Neuroscience (ERCPN) at Maastricht University (ERCPN-176_08_02_2017), the Dutch Medisch-ethische toestingscommissie (METC 20-035; Dutch trial register no. 23432), and by the Deontological Committee of Faculty of Psychology at the University of Lisbon.

## Declaration of competing interest

The authors declare that they have no known competing financial interests or personal relationships that could have appeared to influence the work reported in this paper.

## Data Availability

Data and code can be shared upon reasonable request.

## Appendix A Supplementary data

Supplementary material Image 1
