# Supplementary material for: Multidimensionality of hallucination-like experiences: A factor structure refinement of the Launay-Slade Hallucination Scale
Source: Schizophr Res Cogn. 2025 May 24;41:100368. doi: 10.1016/j.scog.2025.100368 (PMC12152376; doi:10.1016/j.scog.2025.100368)
Supplement: Supplementary file 1 — Supplementary material [file mmc1.docx]

**Supplementary Material**

**Title: Multidimensionality of Hallucination-like Experiences: A Factor Structure Refinement of the Launay-Slade Hallucination Scale**

**Authors:** Honcamp, H.*^a^, Goller, L.K.*^#a^, Amorim, M.^b^, Duggirala, S.X.,^a^ Johnson, J.F.^c^, Schwartze, M.^a^, Pinheiro, A.P.^b^, Kotz, S.A.^a^

*These authors contributed equally to this work

^#^Corresponding author

**Institutional affiliations:**

^a^Department of Neuropsychology and Psychopharmacology; Faculty of Psychology and Neuroscience, Maastricht University, the Netherlands

^b^Centro de Investigação em Ciência Psicológica, Faculdade de Psicologia, Universidade de Lisboa, Lisboa, Portugal

^c^Université Libre de Bruxelles, Belgium

**Contact details of the corresponding author:**

Lisa K. Goller

Dept. Neuropsychology and Psychopharmacology

Maastricht University

Universiteitssingel 40, 6229 ER Maastricht, the Netherlands

E-mail: [lisa.goller@maastrichtuniversity.nl](mailto:lisa.goller@maastrichtuniversity.nl)

ORCID: 0000-0001-6851-7039

**Supplementary Material A.**

**Background information on the Launay-Slade Hallucination Scale**

The Launay-Slade Hallucination Scale [LSHS; 1] is an instrument with satisfactory psychometric properties that is widely used to assess hallucination proneness (HP) in clinical and non-clinical populations [2]. Originally, a 30-item questionnaire with a binary response format (true/false) was administered to non-diagnosed individuals (n = 54), a patient group experiencing auditory hallucinations (n = 42), and a group of prisoners (n = 200). Based on item endorsement frequencies between groups and a factor analysis, rendering a two-factor structure (“Tendency to hallucinatory experiences” and “Negative response set”), the 30 items were reduced to a 12-item questionnaire, establishing the original version of the LSHS.

**Supplementary material B.**

**Supplementary methods**

This section contains additional information on the distribution of LSHS scores across all items, a comprehensive explanation and justification of any methodological deviations from standard practices, as well as technical details about the EFA and factor retention criteria.

**B1. Distribution of LSHS scores across items and response categories**

**Supplementary Table 1.** LSHS item distribution of all response categories (N = 287)

| **Item** | **Frequency (Proportion in %)** | | | | | **Mean** | **SD** |
| --- | --- | --- | --- | --- | --- | --- | --- |
|  | 0 | 1 | 2 | 3 | 4 |  |  |
| 1 | 113 (0.406) | 58 (0.209) | 27 (0.097) | 60 (0.216) | 20 (0.072) | 1.34 | 1.38 |
| 2 | 67 (0.241) | 34 (0.122) | 13 (0.047) | 85 (0.306) | 79 (0.284) | 2.27 | 1.57 |
| 3 | 125 (0.450) | 57 (0.205) | 20 (0.072) | 61 (0.219) | 15 (0.054 | 1.22 | 1.36 |
| 4 | 189 (0.680) | 46 (0.165) | 18 (0.065) | 19 (0.068) | 6 (0.022) | 0.59 | 1.02 |
| 5 | 87 (0.313) | 56 (0.201) | 38 (0.137) | 58 (0.209) | 39 (0.140) | 1.66 | 1.45 |
| 6 | 75 (0.270) | 65 (0.234) | 54 (0.194) | 56 (0.201) | 28 (0.101) | 1.63 | 1.34 |
| 7 | 155 (0.558) | 48 (0.173) | 18 (0.065) | 39 (0.140) | 18 (0.065) | 0.98 | 1.33 |
| 8 | 62 (0.223) | 65 (0.134) | 29 (0.104) | 90 (0.324) | 32 (0.115) | 1.87 | 1.38 |
| 9 | 29 (0.104) | 52 (0.187) | 24 (0.084) | 105 (0.378) | 68 (0.245) | 2.47 | 1.32 |
| 10 | 89 (0.320) | 76 (0.273) | 25 (0.090) | 66 (0.237) | 22 (0.079) | 1.48 | 1.36 |
| 11 | 215 (0.773) | 30 (0.108) | 11 (0.04) | 18 (0.065) | 4 (0.014) | 0.44 | 0.94 |
| 12 | 152 (0.547) | 46 (0.165) | 15 (0.054) | 49 (0.176) | 16 (0.058) | 1.03 | 1.35 |
| 13 | 153 (0.055) | 48 (0.173) | 19 (0.068) | 37 (0.133) | 21 (0.076) | 1.01 | 1.36 |
| 14 | 225 (0.809) | 31 (0.112) | 10 (0.036) | 10 (0.036) | 2 (0.007) | 0.32 | 0.77 |
| 15 | 170 (0.612) | 49 (0.176) | 12 (0.043) | 37 (0.133) | 10 (0.036) | 0.81 | 1.22 |
| 16 | 153 (0.550) | 54 (0.194) | 23 (0.083) | 41 (0.147) | 7 (0.025) | 0.90 | 1.20 |

**B2. Details and justification for EFA parameter choices**

***B2.1 Data suitability for factor analysis***

Although most research using factor analysis relies on Likert scale-based, ordinal variables, EFAs are often conducted using Pearson correlation matrices. However, Pearson correlation assumes continuous, normally distributed data and tends to underestimate the relationship between two ordinal variables [3]. Therefore, it may not adequately represent true data variability. To circumvent this problem, polychoric correlation matrices can be used [3]. EFAs based on polychoric correlations are more robust to non-normality, more consistent with underlying theoretical expectations, and more likely to recover the true underlying factor structure than Pearson correlations [3, 4]. We therefore opted for polychoric correlations in evaluating data suitability for EFA and factor estimation.

Inspection of the polychoric correlation matrix revealed that inter-item correlation coefficients were reasonably high (Supplementary Fig. 1). We observed that one item (item 4; “On certain occasions, I have had the feeling of the presence of someone close who has deceased”) showed generally lower correlations with other items. However, we decided against excluding this item as i) correlation coefficient-based cut-offs guiding item in- and exclusion are rather arbitrary (i.e., a more objective test such as Barlett's test of sphericity should be preferred), and ii) it would limit the comparability with previously obtained EFA results based on all 16 LSHS items.

***B2.2 Factor extraction***

There are two broad families of factor estimation methods: Maximum likelihood (ML) and least squares (LS). Both offer multiple (robust) variants with different distributional assumptions [5]. Most empirical research employing factor analysis relies on ML-based estimators, which are generally preferred for continuous multivariate and normally distributed data. However, it can result in unstable factor solutions for ordinal and non-normal variables [6]. We therefore used the robust mean and variance-adjusted weighted least squares (WLSMV) method for factor estimation, which is recommended for ordinal data, especially when the assumption of multivariate normality is violated [6-8]. Additionally, the robust variant of the WLS estimator is more lenient regarding its sample size requirements than the non-robust variant [3]. Moreover, WLS estimation with polychoric correlations was found to yield better results for non-continuous data as compared to ML-based estimators [9].

***B2.3 Factor retention criteria***

The Kaiser-Guttman Criterion (KGC) has been criticized for being derived from PCA, not EFA, and tending to overestimate the number of factors [10-12]. Additionally, the reliability of the Scree plot was questioned as its interpretation tends to be arbitrary and subjective, i.e., two solutions with e.g., 4 or 5 factors with similar eigenvalues could represent the data equally well. Thus, the Scree plot should rather be used to find a range of values to be considered [13, 14].

**Supplementary Fig. 1.** Polychoric correlation matrix of LSHS items


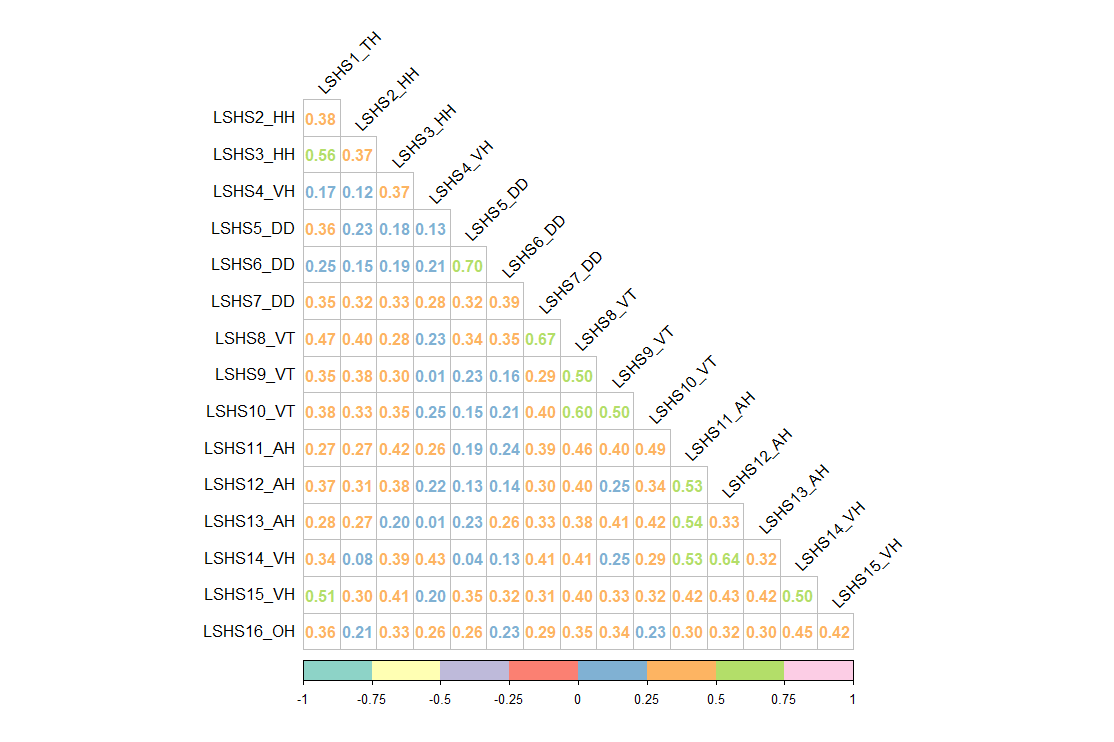


**Supplementary Table 2.** Item-specific KMO values

| **Item** | **KMO** |
| --- | --- |
| 1 | 0.84 |
| 2 | 0.80 |
| 3 | 0.80 |
| 4 | 0.69 |
| 5 | 0.70 |
| 6 | 0.74 |
| 7 | 0.84 |
| 8 | 0.84 |
| 9 | 0.85 |
| 10 | 0.86 |
| 11 | 0.88 |
| 12 | 0.83 |
| 13 | 0.86 |
| 14 | 0.74 |
| 15 | 0.91 |
| 16 | 0.93 |

**Supplementary Fig. 2.** Scree plot


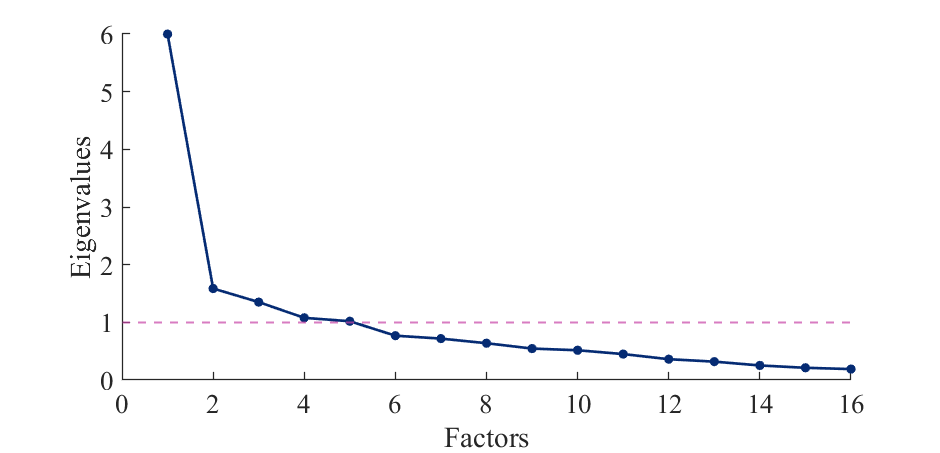


**Supplementary Table 3.** Eigenvalues of all factors prior to factor retention

| **Factor** | **Eigenvalue** |
| --- | --- |
| 1 | 5.99 |
| 2 | 1.59 |
| 3 | 1.35 |
| 4 | 1.08 |
| 5 | 1.02 |
| 6 | 0.72 |
| 7 | 0.72 |
| 8 | 0.64 |
| 9 | 0.55 |
| 10 | 0.52 |
| 11 | 0.45 |
| 12 | 0.36 |
| 13 | 0.32 |
| 14 | 0.25 |
| 15 | 0.22 |
| 16 | 0.19 |

**Supplementary material C. Alternative three- and five-factor solutions**

This section contains two alternatively considered EFA models with three and five factors. LSHS item order was adapted from [15]. Note that the three-factor solution was not supported by the employed factor retention criteria and the five-factor solution was significantly less interpretable due to an increased number of cross-factor loadings (i.e., loadings ≥ | 0.3 | on more than one factor). The four-factor solution was thus deemed most suitable.

**Supplementary Table 4.** Alternative 3-factor EFA model

| **LSHS items** | **F1** | **F2** | **F3** |
| --- | --- | --- | --- |
| 1 | 0.19 | 0.17 | **0.41** |
| 2 | 0.04 | **0.45** | 0.05 |
| 3 | 0.05 | 0.00 | **0.63** |
| 4 | 0.09 | -0.20 | **0.54** |
| 5 | **1.01** | -0.03 | -0.18 |
| 6 | **0.79** | 0.03 | -0.10 |
| 7 | 0.17 | **0.58** | -0.01 |
| 8 | 0.06 | **0.91** | -0.13 |
| 9 | -0.07 | **0.74** | -0.08 |
| 10 | -0.15 | **0.81** | -0.02 |
| 11 | -0.13 | **0.40** | **0.44** |
| 12 | -0.15 | 0.07 | **0.71** |
| 13 | 0.01 | **0.46** | 0.13 |
| 14 | -0.20 | -0.14 | **1.01** |
| 15 | 0.20 | 0.04 | **0.53** |
| 16 | 0.13 | 0.05 | **0.44** |

**Supplementary Table 5.** Alternative 5-factor EFA model

| **LSHS items** | **F1** | **F2** | **F3** | **F4** | **F5** |
| --- | --- | --- | --- | --- | --- |
| 1 | **0.78** | 0.09 | 0.00 | -0.04 | -0.09 |
| 2 | **0.49** | -0.03 | 0.19 | 0.08 | -0.24 |
| 3 | **0.95** | -0.07 | -0.11 | -0.20 | 0.09 |
| 4 | 0.26 | 0.05 | 0.18 | **-0,37** | **0.40** |
| 5 | 0.11 | **0.88** | -0.04 | 0.06 | -0.20 |
| 6 | -0.14 | **0.79** | 0.11 | 0.05 | 0.03 |
| 7 | -0.06 | 0.15 | **0.68** | -0.08 | 0.16 |
| 8 | -0.08 | 0.02 | **0.95** | 0.03 | 0.01 |
| 9 | 0.20 | -0.07 | 0.24 | **0.45** | -0.22 |
| 10 | 0.12 | -0.13 | **0.48** | 0.27 | -0.05 |
| 11 | -0.06 | -0.04 | 0.09 | **0.57** | **0.33** |
| 12 | 0.16 | -0.10 | -0.01 | 0.30 | **0.44** |
| 13 | -0.22 | 0.11 | -0.04 | **0.84** | 0.07 |
| 14 | -0.08 | -0.12 | 0.01 | 0.22 | **0.95** |
| 15 | **0.36** | 0.20 | -0.21 | **0.35** | 0.19 |
| 16 | 0.23 | 0.12 | -0.05 | 0.19 | 0.21 |

**References**

1. Launay, G. and P. Slade, *The measurement of hallucinatory predisposition in male and female prisoners.* Personality and Individual Differences, 1981. **2**(3): p. 221-234.

2. Johns, L.C., *Hallucinations in the general population.* Current psychiatry reports, 2005. **7**(3): p. 162-167.

3. Holgado–Tello, F.P., et al., *Polychoric versus Pearson correlations in exploratory and confirmatory factor analysis of ordinal variables.* Quality & Quantity, 2010. **44**: p. 153-166.

4. Watkins, M.W., *Exploratory factor analysis: A guide to best practice.* Journal of Black Psychology, 2018. **44**(3): p. 219-246.

5. Kyriazos, T. and M. Poga-Kyriazou, *Applied Psychometrics: Estimator Considerations in Commonly Encountered Conditions in CFA, SEM, and EFA Practice.* Psychology, 2023. **14**(5): p. 799-828.

6. Brown, T.A., *Confirmatory factor analysis for applied research*. 2015: Guilford publications.

7. Beauducel, A. and P.Y. Herzberg, *On the performance of maximum likelihood versus means and variance adjusted weighted least squares estimation in CFA.* Structural equation modeling, 2006. **13**(2): p. 186.

8. Goretzko, D., T.T.H. Pham, and M. Bühner, *Exploratory factor analysis: Current use, methodological developments and recommendations for good practice.* Current psychology, 2021. **40**: p. 3510-3521.

9. Barendse, M., F.J. Oort, and M. Timmerman, *Using exploratory factor analysis to determine the dimensionality of discrete responses.* Structural Equation Modeling: A Multidisciplinary Journal, 2015. **22**(1): p. 87-101.

10. Costello, A.B. and J. Osborne, *Best practices in exploratory factor analysis: Four recommendations for getting the most from your analysis.* Practical assessment, research, and evaluation, 2005. **10**(1): p. 7.

11. Howard, M.C., *A review of exploratory factor analysis decisions and overview of current practices: What we are doing and how can we improve?* International journal of human-computer interaction, 2016. **32**(1): p. 51-62.

12. Ledesma, R.D. and P. Valero-Mora, *Determining the number of factors to retain in EFA: An easy-to-use computer program for carrying out parallel analysis.* Practical assessment, research, and evaluation, 2019. **12**(1): p. 2.

13. O’connor, B.P., *SPSS and SAS programs for determining the number of components using parallel analysis and Velicer’s MAP test.* Behavior research methods, instruments, & computers, 2000. **32**(3): p. 396-402.

14. Woods, C.M. and M.C. Edwards, *Essential statistical methods for medical statistics.* Factor Analysis and Related Methods, 2011. **27**(6): p. 174-201.

15. Larøi, F. and M. Van Der Linden, *Nonclinical Participants' Reports of Hallucinatory Experiences.* Canadian Journal of Behavioural Science/Revue canadienne des sciences du comportement, 2005. **37**(1): p. 33.
